# AIMP3 Deletion Induces Acute Radiation Syndrome-like Phenotype in Mice

**DOI:** 10.1038/s41598-018-33303-3

**Published:** 2018-10-09

**Authors:** Doyeun Kim, Sunmi Kim, Youngsun Oh, Songhwa Park, Yoon Jeon, Hongtae Kim, Ho Lee, Sunghoon Kim

**Affiliations:** 1 Medicinal Bioconvergence Research Center, College of Pharmacy, Seoul National University, Seoul, Korea; 2 Division of Convergence Technology, Research Institute National Cancer Center, Goyang, Korea; 3 Department of Biological Science, Sungkyunkwan University, Suwon, Korea

## Abstract

Genomes are mostly protected from constant DNA-damaging threats, either internal or external, which ultimately sustain the organism. Herein, we report that AIMP3, a previously demonstrated tumour suppressor, plays an essential role in maintaining genome integrity in adult mice. Upon induction of the temporal systemic deletion of AIMP3 by tamoxifen in adult mice, the animals developed an acute radiation syndrome-like phenotype, typified by scleroderma, hypotrophy of haematopoietic cells and organs, and intestinal failure. Induction of γH2AX, an early marker of DNA double-strand breaks, was observed in the spleen, intestine, and the highly replicating embryonic cortex. In addition, sub-lethal irradiation of AIMP3 mKO mice dramatically affected organ damage and survival. Using isolated MEFs from conditional KO mice or AIMP3 knockdown cells, we confirmed the presence of spontaneously occurring DNA double-strand breaks by COMET assay and γH2AX induction. Furthermore, γH2AX removal was delayed, and homologous DNA repair activity was significantly reduced. Reduction of RPA foci formation and subsequent Rad51 foci formation probably underlie the significant reduction in homologous recombination activity in the absence of AIMP3. Together, our data demonstrate that AIMP3 plays a role in genome stability through the DNA repair process.

## Introduction

The maintenance of genome integrity is a top priority in living organisms to preserve genetic information across generations. Multiple biological processes exist for optimal responses to the diverse genotoxic stresses from both intrinsic (e.g., DNA replication) and extrinsic sources (e.g., radiation). Various disorders, including Bloom syndrome, ataxia telangiectasia, Fanconi anaemia, and Nijmegen breakage syndrome, are derived from deficiencies in DNA repair^1^. Common clinical presentations include defects in the haematopoietic system, radiosensitivity in the skin, and predisposition to cancer, such as polyposis in the colon^2,3^. Genetic mouse models for candidate genes have provided strong evidence for the validation of these disorders, as well as powerful tools for *in vivo* studies. However, the essential nature of the machinery involved in genome integrity further complicates functional study under physiological conditions. Therefore, conditional manipulation of genetic mouse models is required, as demonstrated in ATR and NBS mouse models^4,5^.

Acute radiation syndrome (ARS) is caused by excessive exposure to a high-level radiation source. Haematopoietic, gastrointestinal, and/or cutaneous symptoms are common in ARS, highlighting the heightened sensitivity of rapidly dividing cells^6^. Highly replicating cells in developing organs are more vulnerable to DNA damage because these cells are subject to more replication stress. Prolonged replication fork stalling leads to the collapse of the repliosome, which generates double-strand breaks^7^. Homologous recombination (HR), which is preferably active compared to the error-prone non-homologous end joining (NHEJ) during replication, repairs the collapsed fork in an error-free manner^8,9^. The importance of homologous recombination to haematopoiesis and development are well known due to human chromosome instability syndromes^7^. γH2AX, a phosphorylated form of histone, recruits repair factors and activates checkpoint signalling^10^. γH2AX rapidly disappears from the bone marrow or CHO cells upon irradiation, implying high innate repair activity^11^. The absence of HR repair factors, including CtIP, Recq1, or Srs2, leads to increased toxic inter-HR intermediates or spontaneously accumulating γH2AX, likely due to loss of HR repair resolution^12–14^. In addition, ATR-Seckel mice and HELQ knockout mice provide strong evidence of the replicative stress-mediated DNA damage response *in vivo*^15,16^. Complex networks of DNA damage signalling and repair in replicating cells are not yet clearly understood.

AIMP3 is a scaffolding protein that constitutes a multisynthetase complex^17,18^. In addition to increasing the efficiency of Met-tRNA charging^19^, a role for AIMP3 in the activation of the DNA damage response pathway and the suppression of oncogene-mediated transformation has been previously described^20,21^. Of note, heterozygous AIMP3 animals develop tumourigenesis of haematopoietic organs in late stages of life^20^. The homozygous loss of AIMP3 results in early embryonic lethality, hindering further investigation of its *in vivo* function. To better understand the physiological role of AIMP3, we generated conditional knockout mice to systemically eliminate AIMP3 upon tamoxifen treatment. Our results illustrate that the consequences of AIMP3 deletion include phenotypic recapitulation of ARS, which affects highly dividing tissues, including haematopoietic organs and intestines. Spontaneous DNA strand breakage and reduction of DNA repair activity indicate that AIMP3 contributes to the resolution of DNA damage. The loss of AIMP3 accelerates the response to ARS *in vivo*, emphasizing its protective role against acute radiation toxicity.

## Results

### Tamoxifen-induced systemic deletion of AIMP3 in the adult mouse induces haematopoietic and intestinal failure

**Figure 1:**
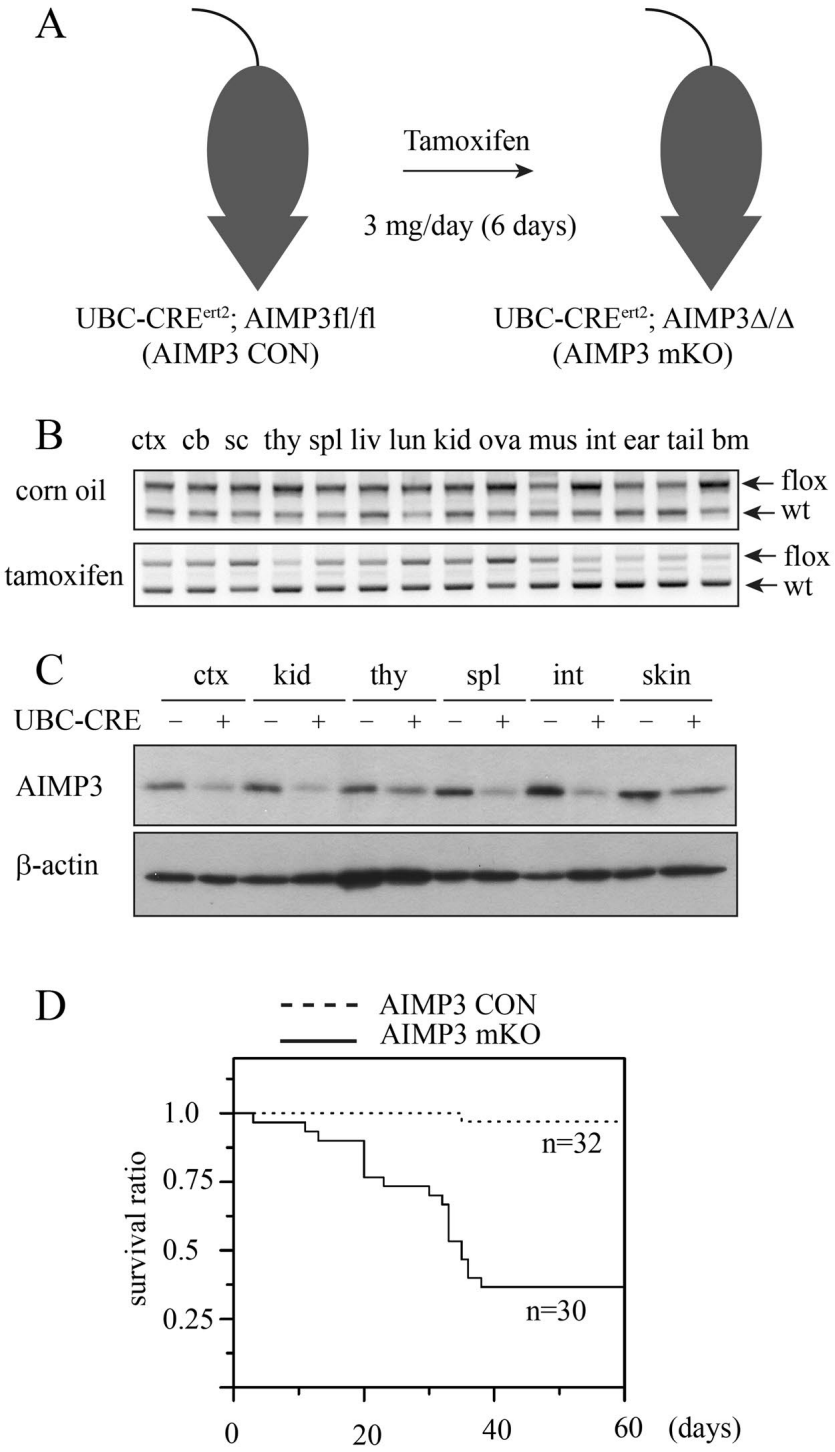
AIMP3 is a critical survival factor in adult mice. (**A**) Schematics of tamoxifen-mediated genomic deletion of AIMP3 in adult mice. Tamoxifen (3 mg/day) was administered intraperitoneally to UBC-CRE^ert2^; AIMP3^fl/fl^ mice for six days to induce systemic deletion of AIMP3. (**B**) Genomic PCR of AIMP3 gene locus flanking the frt-loxP sequence from corn oil-treated (top) or tamoxifen-treated (bottom) UBC-CRE^ert2^; AIMP3fl/+ mice. The ratio between PCR bands from floxed allele (with frt-loxP sequence; flox) and wild-type (wt) was decreased in multiple organs. Ctx: brain cortex, Cb: cerebellum, sc: spinal cord, thy: thymus, liv: liver, lun: lung, kid: kidney, ova: ovary, mus: muscle, int: intestine, bm: bone marrow. (**C**) Immunoblotting of AIMP3 in multiple organs of AIMP3 mKO. Tissue lysates were prepared three weeks after tamoxifen treatment. AIMP3fl/fl mice without UBC-Cre transgene were used as a control. Lysates were immunoblotted with anti-AIMP3 or anti-β-actin antibodies. β-actin level is shown as a loading control. Representative results from duplicate experiments are shown. (**D**) Survival curve of AIMP3 mKO mice upon tamoxifen treatment. The survival of mice was monitored from the first date of tamoxifen injection (Day 0). AIMP3fl/fl mice without UBC-Cre were also tamoxifen-treated and used as a control. More than 60% AIMP3 mKO died within 40 days of the first injection (p < 0.001 by log-rank test).

We targeted the AIMP3 locus to generate conditional alleles with loxP flanking exon 2 and exon 3. Correct targeting was confirmed by Southern blot and PCR analysis (Fig. S1). For time-controlled AIMP3 deletion in adult mice, we crossed AIMP3 floxed mice with UBC-CRE^ert2^ mice to generate AIMP3 fl/fl; UBC-CRE^ert2^ mice. To maximize the efficiency of the AIMP3 deletion, we repeatedly administered tamoxifen by intraperitoneal injection for six days (Fig. 1A and Materials and Methods). Because temporal deletion of the locus in adult mice is not complete, we designated tamoxifen-treated conditional AIMP3 mice as AIMP3 mKO, meaning mosaic knock-out^22^. To assess the effect of UBC-CRE^ert2^-mediated deletion of the AIMP3 floxed locus, we performed genomic PCR flanking the loxP sequence. In corn oil-treated heterozygous mice, the ratio between the floxed (upper band) and wild-type (lower band) alleles was 1 or higher, whereas in tamoxifen-treated heterozygous mice, it decreased to less than 0.5 in most organs (Fig. 1B). We confirmed the decrease of AIMP3 at the protein level from several organs. Most tissues exhibited greater than 50% reduced expression, and we consistently observed the highest reduction in the spleen and intestines (Fig. 1C).

To investigate the effect of AIMP3 deletion in mice, we set up two groups, AIMP3 fl/fl without UBC-CRE^ert2^ (AIMP3 CON) and AIMP3 fl/fl with UBC-CRE^ert2^ (AIMP3 mKO). Tamoxifen was given to all mice 8–10 weeks old in both groups to exclude potential side effects of the drug. All mice were observed for gross phenotype, including weight loss, fur state, and locomotive behaviour twice a week. AIMP3 mKO mice gradually lost weight, becoming less active and moribund. The viability of these mice was severely affected during the two months after tamoxifen treatment, and major losses occurred between 20 and 40 days (Fig. 1D). Less than 40% of AIMP3 mKO mice survived the treatment period and did not experience challenges in the life span, presumably due to the repopulation of the AIMP3-containing cells. Approximately 30% of sick mice also exhibited a skin phenotype, consisting of hair loss and thickening of skin around the rostrum and limbs (Fig. S2A). In mild cases, only a loss of sebaceous gland cells was observed, whereas in severe cases, the epithelium and epidermis were thickened and had become infiltrated by macrophages (Fig. S2B).

**Figure 2:**
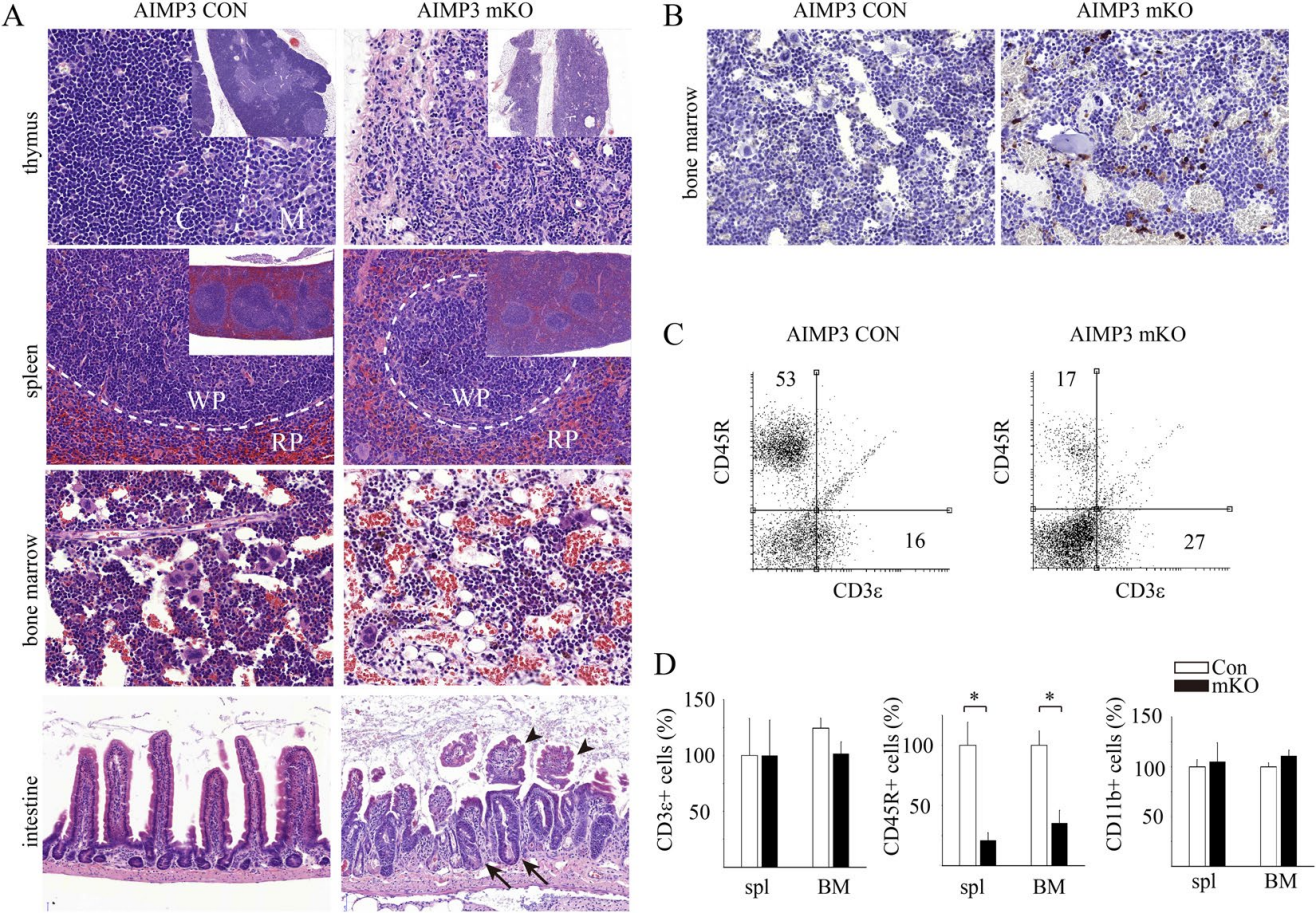
Haematopoietic and gastrointestinal failure in AIMP3 mKO mouse. (**A**) Hypotrophy of haematopoietic and intestinal organs in AIMP3 mKO. At day 30, the thymus, spleen, bone marrow and intestine from AIMP3 CON (left column) and AIMP3 mKO (right column) were isolated, and tissue sections were stained with haematoxylin-eosin (HE). The inset shows a low-resolution image of the HE-stained tissue. Dotted lines mark the boundary between the cortex (C) and medulla (M) in the thymic lobe and between the white pulp (WP) and red pulp (RP) in the spleen, respectively. Arrows and arrowheads in the intestine indicate elongated crypts and blunted villi, respectively, in AIMP3 mKO mice. (**B**) Bone marrow isolated from AIMP3 CON and AIMP3 mKO mice were immunostained with antibodies against cleaved caspase-3. (**C**) Splenocytes from AIMP3 CON (left) and AIMP3 mKO (right) mice were analysed by FACS cytometry using the markers CD3ε (pan-T cells) and CD45R (pan-B cells). The number indicates the percentage of the cells in the quadrant. Representative results from three animals are shown. (**D**) CD3ε–(left), CD45R- (middle) and CD11b- (right) positive cells were analysed by FACS in spleen and bone marrow from AIMP3 CON and AIMP3 mKO mice. Three to five animals per group were used. Error bars indicate standard error. Asterisks denote significant difference between groups by Student’s t-test (*p < 0.01).

To determine the phenomena underlying survival defects at the tissue level, we performed necropsy on affected mice and matched controls. Among analysed tissues, we consistently observed remarkable reduction in the gross size of the thymus and spleen (Fig. S2C). In addition, histological analysis of haematopoietic organs revealed structural defects (Fig. 2A). Immune cell-enriched cortex in the thymus and white pulp in the spleen were either missing or greatly shrunken in AIMP3 mKO mice. The density of immune cells was decreased, and the infiltration of red blood cells was increased in AIMP3 mKO bone marrow. In addition to haematopoietic organs, the intestine was also consistently affected in AIMP3 mKO mice. In comparison with control intestine showing elongated villi and shallow crypts, in AIMP3 mKO intestine, villi were shortened or blunted and crypts had elongated (Fig. 2A). The phenotype was widespread throughout the entire intestine. Furthermore, affected tissues from AIMP3 mKO mice exhibited higher numbers of pyknotic nuclei compared to wild-type tissues (data not shown). We observed many cleaved caspase 3-positive cells in the bone marrow of AIMP3 mKO, but not in wild-type tissues, suggesting that apoptotic cell death underlies the degenerative phenotype (Fig. 2B). The quantitation of cleaved caspase 3-positive cells confirmed a significant difference between control and mKO tissues (4 ± 1 and 127 ± 10/mm^3^, respectively, p = 0.0029 by Student’s t-test).

We further evaluated haematopoietic organ failure using FACS analysis of spleen and bone marrow cells from AIMP3 mKO mice. We analysed the spleen with CD3ε (pan-T cell marker), CD45R (pan-B cell marker), and CD11b (pan-macrophage marker) and found that CD45R-positive cells were significantly decreased in AIMP3 mKO (Fig. 2C). We also observed a significant reduction of CD45R-positive cells in bone marrow and peripheral blood, whereas the numbers of CD3ε– or CD11b-positive cells were not significantly different (Figs 2D and S2D). The total numbers of red blood cells and white blood cells (WBC) in peripheral blood was not significantly different between AIMP3 mKO and control mice (Fig. S2D).

Together, our results demonstrate a critical necessity for AIMP3 in the survival of mice, likely through maintaining the haematopoietic, especially the B cell population, and intestinal systems.

### Spontaneous DNA strand breakage by AIMP3 deletion in cells

**Figure 3:**
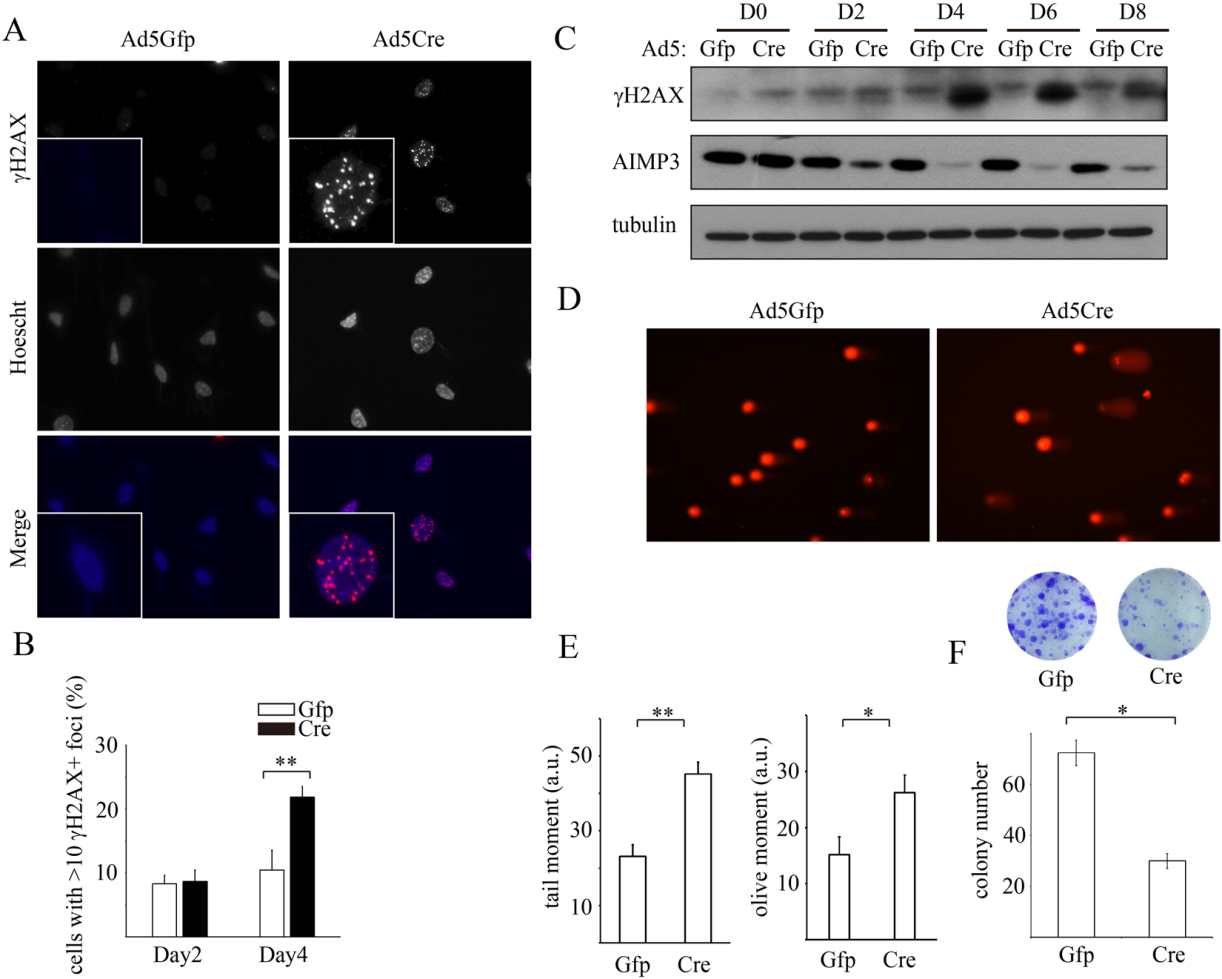
DNA strand breakage spontaneously arises in response to genetic deletion of AIMP3. (**A**) Immortalized AIMP3 fl/fl MEFs were treated with Ad5Gfp (control) or Ad5Cre virus for four days and immunostained with antibodies against γH2AX. (**B**) γH2AX positive cells were quantified two and four days after the indicated virus treatment. More than 200 cells were observed from four independent fields. Two-way ANOVA with Bonferroni’s posttests was performed for statistical analysis. (**C**) AIMP3 fl/fl MEFs were harvested at indicated times after virus treatment. Cell lysates were analysed for the protein levels of γH2AX and AIMP3 by immunoblotting. Tubulin-α level was used as a loading control. (**D**,**E**) Occurrence of DNA breakage was analysed by Comet assay in virus-treated AIMP3 fl/fl MEFs. (**D**) Representative images are shown from 20–25 randomly selected fields. (**E**) The olive and tail moment was calculated using the Open comet plugin in the ImageJ program. Statistical significance was analyzed by Student’s t-test. (**F**) Virus-treated MEFs were plated at low density for a colony-forming assay (as described in Materials and Methods). Colony numbers from three independent experiments were quantified, and representative images are shown. Statistical significance was analyzed by Student’s t-test. Error bars indicate standard error. * and ** denote p < 0.05 and p < 0.01, respectively.

Defects in haematopoietic organs and the gastrointestinal tract are well known in radiation-mediated biological responses. In addition, we reported the function of AIMP3 in maintaining genomic stability^21^. To determine whether the loss of AIMP3 is sufficient for the generation of the DNA damage response, we induced genomic deletion of AIMP3 in MEF cells isolated from AIMP3 fl/fl mice using Ad5Cre virus infection. In Ad5Cre-treated MEFs, but not in Ad5GFP-treated cells, we observed the accumulation of γH2AX in the nuclei (Fig. 3A). Four days after virus treatment, γH2AX-positive cells constituted approximately 20% of the total cells (Fig. 3B). Decreased AIMP3 and accumulation of γH2AX were also confirmed by immunoblotting. The protein levels of AIMP3 were decreased to minimal levels by day four, and simultaneously, we observed obvious induction of γH2AX (Fig. 3C). To directly measure DNA strand breaks in response to loss of AIMP3, we also performed a COMET assay. Cells with DNA breakage significantly increased upon Ad5Cre treatment (Fig. 3D,E). Consistently, DNA breakage at the chromosomal level was also observed at higher abundance in micronuclei-containing cells in AIMP3 deletion cells (Fig. S3A). Occurrence of double strand breakage was further confirmed by DSB specific neutral COMET assay (Fig. S3B). We performed colony-forming assays to indirectly measure the genotoxic effect of AIMP3 loss and observed that the colony-forming ability decreased to one-third that of control cells (Fig. 3F).

### Defective homologous recombination repair in AIMP3 knockdown cells

**Figure 4:**
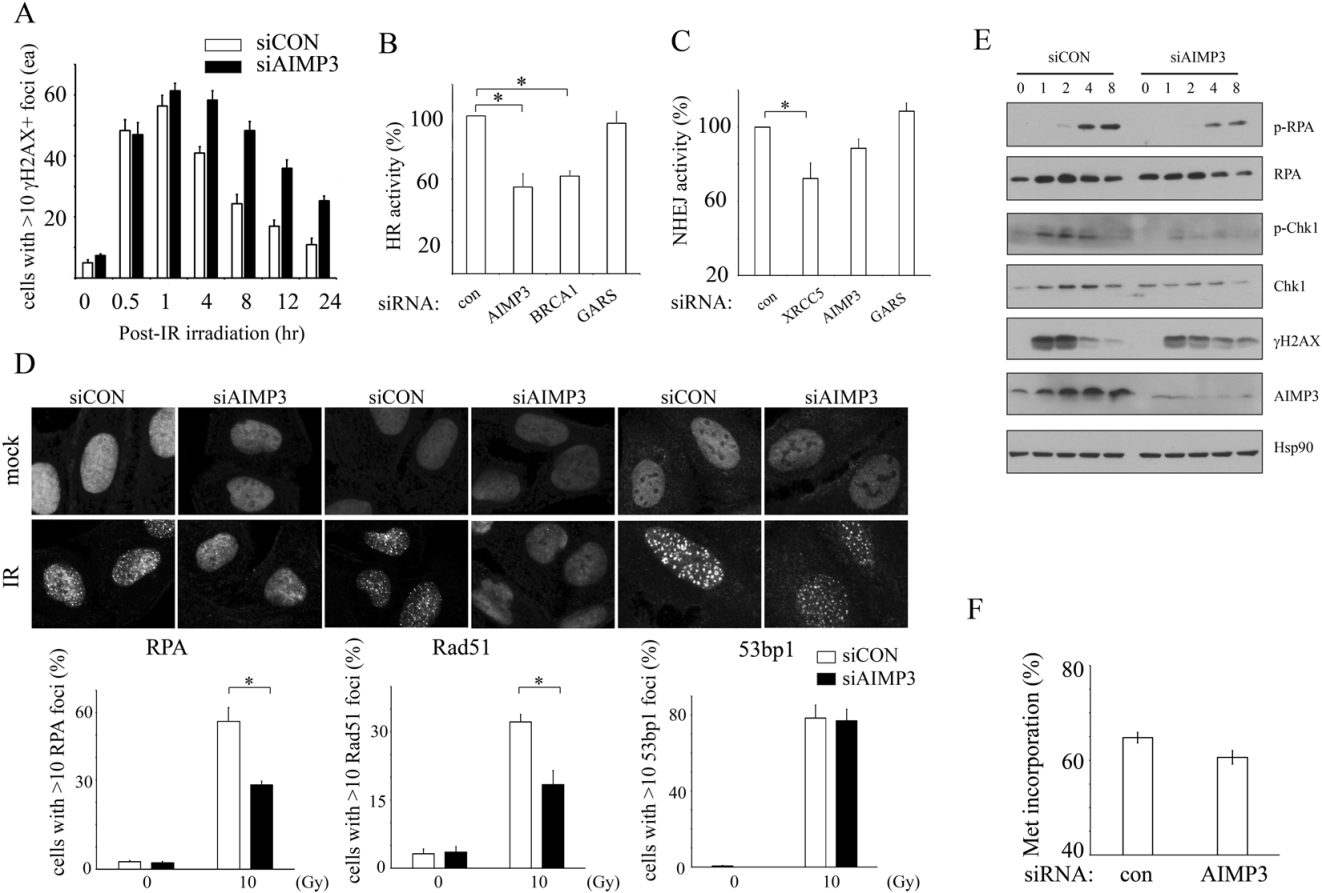
Homologous recombination activity is reduced in AIMP3-deficient U2OS cells. (**A**) Kinetics of γH2AX foci formation in AIMP3 KD U2OS cells. SiRNA-treated U2OS cells were immunostained with γH2AX antibody. γH2AX-positive cells were counted from four independent fields (p < 0.001 by two-way ANOVA test). (**B**,**C**) Measurement of homologous recombination activity (**B**) or non-homologous end-joining activity (**C**) in U2OS cells treated with siCON, siAIMP3, siBRCA1, siXRCC3 or siGRS. Percentages of GFP(+) cells among 10,000 U2OS cells are presented by FACS analysis. Three independent experiments were performed for statistical analysis. *Notes significant difference at p < 0.05 by one-way ANOVA with dunnett’s test. (**D**) Measurement of foci formation by immunostaining using RPA (left), Rad51 (middle), and 53BP1 (right) antibodies. SiRNA-treated U2OS cells were irradiated (10Gy), and foci were observed at 16, 2, and 4 hr after irradiation, respectively. Nuclei with more than 10 foci were counted as positive and quantified as a percentage of total cells. More than 200 cells from four independent fields were observed (*p < 0.001 by two-way ANOVA with Bonferroni’s posttests). (**E**) Measurement of RPA and Chk1 phosphorylation levels in AIMP3 KD U2OS cells. SiCON or siAIMP3 treated U2OS cells were irradiated, and total protein was isolated at indicated time points. Lysates were immunoblotted with indicated antibodies. Hsp90 was used as a loading control. (**F**) Protein synthesis rate in siAIMP3 treated cells was measured by [^35^S] methionine incorporation into total protein. Results were obtained from triplicate samples. Error bars indicate standard error.

According to a previous report, AIMP3 mediates the activation of ATM/ATR and the downstream target p53, suggesting its role in the initiation of DNA damage signalling. When we assessed IR-induced DNA damage foci formation in AIMP3 deletion cells, we saw no evidence of defects in foci formation, including in MDC1, RAP80, or γH2AX (Fig. S4). Mouse models with defects in the DNA repair machinery have shown that the genomic instability is derived from replicative stress^7^. We hypothesized the function of AIMP3 is required for DNA repair machinery. Upon low-dose IR exposure in control cells, γH2AX-positive cells dramatically increased and gradually decreased after one hr to basal levels over 24 hr. In contrast, the gradual decrease of γH2AX-positive cells was much slower in AIMP3 knockdown cells than in control cells, although initial formation of γH2AX foci was similar (Fig. 4A). We tested the specific role of AIMP3 in DNA repair using the homologous recombination assay because it is a major repair event during replication. Cells with reduced AIMP3 by siRNA treatment have shown decreased activity of homologous recombination, as low as that of BRCA1 knockdown cells. In contrast, treatment of control cells with siGARS showed comparable activity to control (Fig. 4B). However, non-homologous end-joining (NHEJ) activity was not significantly changed in AIMP3 knockdown cells (Fig. 4C). The specific reduction of gene expression by siRNA was confirmed by RT-PCR (Fig S5). HR and NHEJ activity were measured by FACS analysis using stable U2OS cells transfected with pDRGFP or pEJ5 (see Materials and Methods).

Because RPA associates with single-strand DNA after resection to direct homologous recombination processes^9^, we assessed whether foci formation was affected in AIMP3 knockdown cells. RPA foci-positive cells were significantly reduced in AIMP3 knockdown cells, and consequently, Rad51 foci replacement was also reduced (Fig. 4D). In contrast to RPA and Rad51, 53bp1 guides NHEJ and competes with HR response. We found that 53bp1 foci formation was not affected, consistent with no significant changes occurring in NHEJ activity. Delayed or reduced levels of RPA phosphorylation was confirmed by immunoblotting (Fig. 4E). Chk1 phosphorylation was also slightly reduced, indicating RPA-mediated ATR activation was compromised in the absence of AIMP3. We observed similarly decreased RPA responses, as well as HR defects in AIMP3 KO MEFs treated with Ad5Cre (Fig. S6A,B). These results suggest that AIMP3 is involved in the HR but not in the NHEJ response upon DNA double-strand break damage, such as from ionizing radiation.

To rule out the effect on global translation as coming from an MSC scaffold protein, methionine incorporation was assessed in the same set of cells used in the HR assay. There was no significant difference between control and AIMP3 knockdown cells (Figs 4F and S6C).

Taken together, our results suggest that AIMP3 plays a critical role in HR repair via the recruitment of RPA and Rad51.

### DNA damage induction in AIMP3 mKO mice

**Figure 5:**
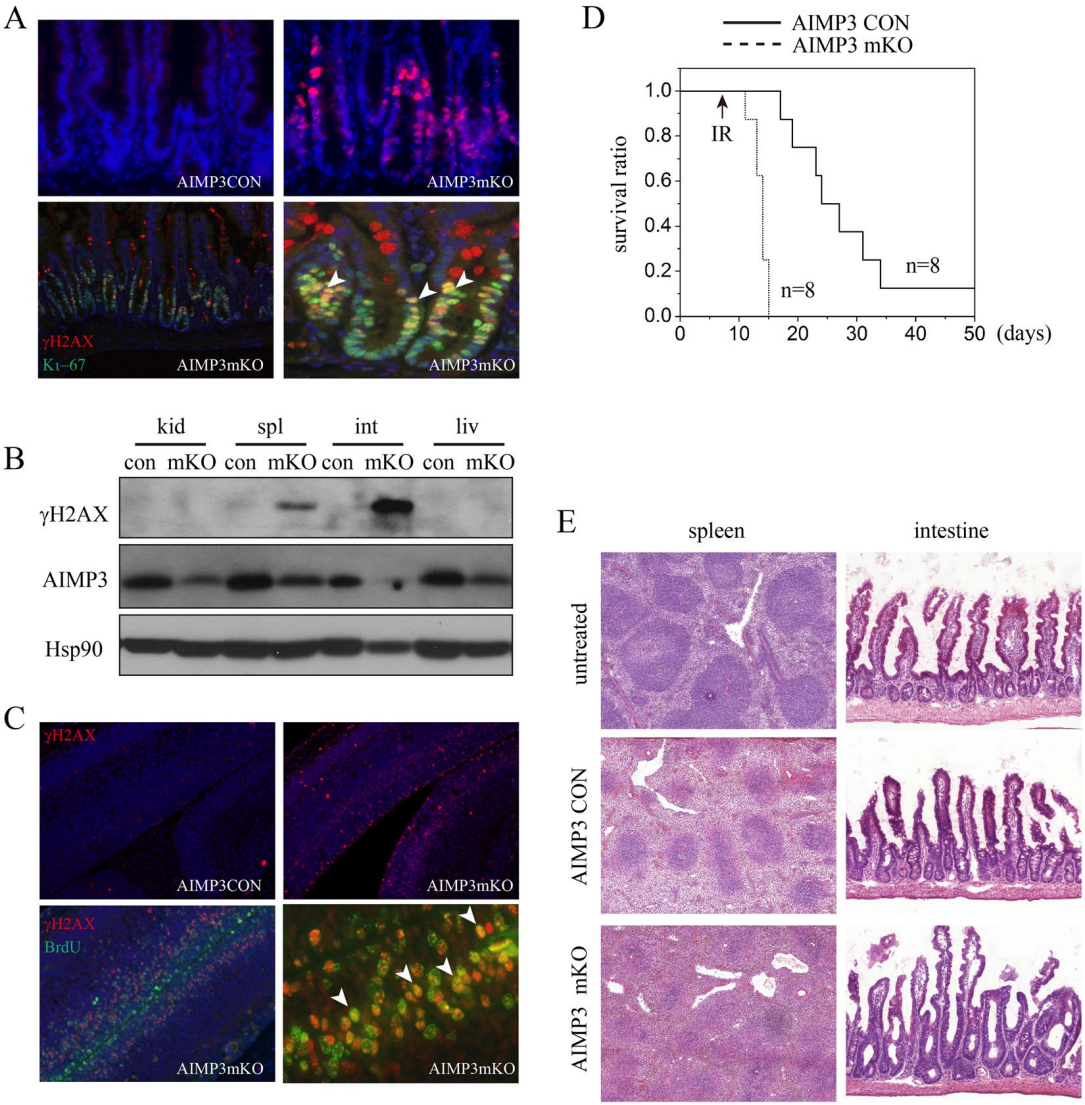
DNA strand breakage in AIMP3 mKO mice/embryos and increased sensitivity to IR. (**A**) Intestines from AIMP3 control and mKO mice were immunostained with antibodies against γH2AX (top) or co-stained with antibodies against Ki-67 (bottom). Arrowheads indicate cells with double staining. (**B**) Lysates from indicated tissues were analysed for levels of γH2AX and AIMP3 by immunoblotting using indicated antibodies. Hsp90α/β was used as a loading control. (**C**) Cortical brain sections from AIMP3 CON and mKO embryos were immunostained with antibodies against γH2AX (top) or co-stained with antibodies against BrdU (bottom). BrdU was injected into pregnant females for labelling of dividing cells in the embryonic cortex. Arrowheads indicate cells with double staining. (**D**) Survival curve of AIMP3 mKO mice in response to ionizing radiation. Ionizing radiation (7 Gy TBI) was administered on Day 8. The survival of mice was monitored from the first date of tamoxifen injection (Day 0). All AIMP3 mKO mice died within a week after irradiation (p < 0.001 by log-rank test). (**E**) Histology of irradiated AIMP3 mKO mice. Spleens and intestines were obtained from AIMP3 CON and AIMP3 mKO three days after irradiation, and tissue sections were stained with H&E (n = 2).

We further evaluated induction of DNA strand breakage in mouse tissue. We performed immunostaining of γH2AX in AIMP3 mKO tissue sections. Affected organs, including intestines and spleen, exhibited higher incidence of γH2AX-positive cells (Fig. 5A). In the intestine, γH2AX-positive cells were located in the elongated crypt region, where a high number of proliferating cells reside. When we performed double-staining using Ki67, a marker for proliferating cells, we observed that the majority of cells were co-immunostained with both markers (Fig. 5A). Induction of γH2AX in tissue lysates was also confirmed by immunoblotting. Induction was obvious in severely affected organs, including the spleen and intestine, but not in the kidney or liver (Fig. 5B).

To rule out possible induction of DNA strand break secondary to a complex catastrophic event *in vivo*, we examined spontaneous induction of γH2AX in the context of mouse development. Sections from E13.5 embryos of AIMP3 mKO were immunostained with γH2AX. Ventricular and subventricular regions of brain cortex, where cell proliferation is high, exhibited obvious increases in γH2AX immunoreactivity. In addition, to determine whether proliferating cells in those regions are positive for γH2AX, we labelled proliferating cells by injecting BrdU and co-immunostained with the antibody against BrdU. Double-positive cells were enriched in the subventricular zone (Fig. 5C).

We further confirmed DNA damage response *in vivo* by examining whether loss of AIMP3 preserves or accelerates cell survival in response to *in vivo* irradiation. For this, we exposed AIMP3 Con and AIMP3 mKO mice to a sub-lethal dose (7 Gy) of total body irradiation and observed the survival ratio in the colonies. All irradiated AIMP3 mKO mice became moribund and died within a week after irradiation, whereas AIMP3 Con mice gradually died over the course of one month after irradiation, and one mouse survived irradiation (Fig. 5D). We also observed defects in the spleen and intestine by histology. The size of the white matter in the spleen was dramatically reduced, and blunted villi and elongated crypts in the intestine were further elevated in tamoxifen-administered animals (Fig. 5E). Potentiation of radiation damage response in AIMP3 mKO mice suggests that AIMP3 is crucial for protecting against acute DNA damage.

Together, our results indicate that loss of AIMP3 is sufficient to induce spontaneous DNA strand breakage. Induction of γH2AX in the proliferating zones suggests a protective role of AIMP3 during DNA replication.

## Discussion

Our previous finding on AIMP3 demonstrated its function in responding to extrinsic DNA damaging agents and oncogenic signals, which include translocation to the nucleus^20,21^. In contrast, herein, we report *in vivo* evidence that AIMP3 is required to maintain genome integrity under physiological conditions, especially in organs with highly replicating cells. Our conditional mouse model spontaneously developed a strong acute radiation syndrome (ARS) phenotype over a short-term window, demonstrating the functional importance of AIMP3 in genomic integrity.

Although AIMP3 is a scaffold protein for the MSC complex, unlike other members of this complex, it is a crucial factor during early development^20^. To study the significance of AIMP3 in multiple biological perspectives, we generated mice in which we induced deletion of the gene after early development. Temporal deletion of AIMP3 in adult mice resulted in development of organ failure in organs with high mitotic indices, including haematopoietic, gastrointestinal, and epidermal structures (Fig. 2). Tissues from these mice revealed cells with spontaneously generated double-strand breaks, likely due to impaired homologous recombination in the absence of AIMP3 (Figs 3 and 4). In addition, AIMP3 deletion potentiated radiosensitivity of mice, resulting in acute death with profound tissue damage (Fig. 5).

It is worth to note rather specific B lymphocyte reduction in AIMP3 KO mice. It phenocopies acute radiation syndrome shown in mouse model, in which lymphocytes including B or T cells are more sensitive than the differentiated cells such as macrophages, dendritic cells and granulocytes^23^. Perhaps, the reason for lymphocyte specificity is due to the antigen-induced cell proliferation which activates checkpoint signaling in the presence of DNA damage. B lymphocytes might be more vulnerable to AIMP3 deletion because they have additional source of intrinsic DNA damage. B lymphocytes undergoes recombination processes including class switching and somatic hypermutation by activation-induced deaminase (AID) in response to antigen exposure^24–26^. Thus, the intrinsic recombination processes would additionally contribute to innate DSB stress unless they are properly controlled.

There is a critical balance between HR and NHEJ for proper somatic hypermutation^27^. In addition, HR prevents AID-mediated widespread genomic breaks during this process^28,29^. Because early markers of DNA damage foci formation in AIMP3 CKO MEFs and KD cells were not affected (Fig. S4), it seems that AIMP3 plays a role in downstream events to facilitate DSB repair efficiency. AIMP3 is a small scaffold protein with a single GST homology domain; therefore, it is tempting to speculate its role in recruitment of the repair complex, such as CtIP, one of the major nucleases in B lymphocytes that directs HR in providing stretches of ssDNA^30^.

Intestines are one of the organs with high rates of spontaneous HR events^31^. Interestingly, crypt base columnar (CBC) stem cells are highly resistant to radiation compared to transit amplifying (TA) cells due to their differing mechanistic functions during HR^32^. Our results also revealed that spontaneous induction of γH2AX in the intestines preferentially occur in the TA zone, not in the CBC zone, suggesting that AIMP3 plays a key role in protecting genomic integrity in the TA zone. Consistently, when sub-lethal irradiation was administered to AIMP3 mKO mice, the response was significantly potentiated, indicating that AIMP3 functions in the protection of radiation-mediated DSB repair. Haploinsufficiency of Rad21, a key regulator of the cohesion complex, revealed its involvement in acute radiation toxicity and hypersensitivity in the intestine due to impaired HR function^33^. Cohesion antagonizes γH2AX spreading, and the absence of cohesion increases γH2AX level, especially in spontaneous DSBs during replication^34–36^. Because AIMP3 induction and translocation to the nucleus are observed in a cell cycle-dependent manner in colon carcinoma cells, it would be interesting to determine whether AIMP3 shares this functional pathway with the cohesion complex^20^.

Replication fork stalling is one of the major sources of replication stress^37,38^. RPA-coated ssDNA recruits both the 9-1-1 complex and ATR to suppress new origin firing and activates cell cycle checkpoints. Defects in embryogenesis, as well as severe cell loss in haematopoietic, reproductive, and intestinal organs, are hallmarks of ATR mK and ATR-Seckel mice^4,15,39–41^. Acute cell loss in tissues with continuous cell proliferation suggests the protective role of ATR in replicative stress^15,42^. Absence of ATR or the hypomorphic allele (Seckel) generates γH2AX foci in highly proliferating cells, leading to organ failure and supporting the importance of the control of fork stress^15,43^ Close resemblance between ATR mKO and AIMP3 mKO strongly suggests that AIMP3 plays a role in the regulation of replicative stress.

During prolonged replication stress, the repliosome collapses and a new replication origin fires, resulting in double-strand breaks^44^. The MRN complex and ATM phosphorylate DNA strand breaks (γH2AX) from the collapsed fork and repairs primarily by homologous recombination (HR). ATM KO mice exhibit chromosome fragmentation and immune defects. It is also noteworthy that the inducible null murine model of NBS1, a key player of homologous recombination in the MRN complex, also displays lymphatic tissue defects^5^. Delayed removal of γH2AX and reduced HR repair activity in AIMP3 KD cells suggest involvement of AIMP3 in HR-mediated DNA damage repair (Fig. 4A,B,E). In addition, RPA foci formation and Rad51 replacement, which are critical processes in HR, were reduced in AIMP3 KO MEFs and KD cells (Figs 4 and S4). Our present results suggest a possible role of AIMP3 in homologous recombination. Therefore, AIMP3 controls genomic integrity by reducing double-strand breaks derived from replicative stress.

Decreased tolerance to ionizing radiation is a common feature in DNA repair deficiency. Deficiency of Ku80, DNA-PKcs, BRCA or FANCD2 in either mice or patients results in enhanced radiation sensitivity. Interestingly, loss of p53, a well-studied checkpoint control molecule, makes mice highly resistant to haematopoietic failure but more sensitive to GI syndrome^45,46^. On the other hand, deficiency of p53 activator, ATM or ATR, severely affects the thymus, and haematopoietic failure was enhanced in combined deletion with p53, differentiating the underlying mechanism of p53 and ATM or ATR in genome instability. Assuming that p53 is not activated by radiation in the absence of AIMP3, the potentiation of radiation damage in AIMP3 mKO suggests a differing repair pathway distinct from p53. Our results support the idea that AIMP3 not only functions through p53 but also functions independently through DSB-HR to reduce damage from radiation.

The importance of targeting tumours by sensitizing them to radiation or protecting sensitive organs, such as those in the haematopoietic and gastrointestinal systems, from radiation is becoming more appreciated. In this respect, Gurung *et al*. recently reported AIMP3 expression in tumours as a beneficial prognostic marker of radiotherapy in a certain form of bladder cancer^47^. In addition, AIMP3 variants are associated with high DNA damage levels in a Chinese population, although characteristics of those variants are not yet known^48^. Our study, on the other hand, demonstrates the loss of AIMP3 in tumours as a beneficial factor for DNA-damaging anticancer treatments, insinuating that AIMP3 itself as a potential target against the genomic integrity of cancer cells. Tight regulation of AIMP3 levels might be necessary to ensure proper function. Considering its complicated contribution to the DNA damage response, careful study should be approached in a context-dependent manner.

## Methods

### Generation of AIMP3 conditional knockout mice

A 15-kb DNA fragment containing exons 1, 2, 3 and 4 of the murine AIMP3 gene was retrieved from BAC clones (bMQ-308J16) into a pBluescript phagemid system, as described in a previously reported procedure^49^. Generation of targeted ES cell clones and germline transmission of the AIMP3^puro^ allele were performed as previously described^50^. AIMP3^puro/+^ mice were crossed with FLP mice to generate AIMP3^fl/+^ without the selection cassette. The floxed allele was genotyped using primers, AIMP3fl F: 5′-TCC TTG CTC CTT GCA CTT TT-3′ and AIMP3fl R: 5′-TTC AGA CAC CCC AGA AGA GG-3′, which generate a longer product than wild-type. AIMP3^fl/fl^ mice were crossed with UBC-Cre^ERT2^ mice (Jax Stock# 008085) to generate UBC-Cre^ERT2^; AIMP3^fl/fl^. UBC-Cre^ERT2^; AIMP3^fl/+^ or AIMP3^fl/fl^ mice without Cre were used as controls. For genotyping the Cre transgene, we used the follow primer set: Cre F: 5′-TCT CTG ACC AGA GTC ATC CTT AGC-3′, Cre R: 5′-TAA AGA TAT CTC ACG TAC TGA CGG TG-3′. Tamoxifen (3 mg/ml in corn oil) was administered via serial intraperitoneal injections for six days. For analysis at embryonic day 13.5, tamoxifen was injected into the pregnant female at E11.5 and E12.5. Deletion of the floxed gene was confirmed by PCR analysis of loxP sites using genotyping primers. PCR products from control and mKO mice were analysed using 1.5% gels separately to compare deletion efficiency. Deletion products were detected using primers, AIMP3fl del: 5′-GTC CAG TAA GCC ACC CAA AA-3′ and AIMP3fl R. This study was reviewed and approved by the Institutional Animal Care and Use Committee (IACUC) of the National Cancer Center Research Institute and Seoul National University. We confirm that all experiments were performed in accordance with relevant guidelines and regulations.

### Survival analysis of the AIMP3 conditional knockout mouse

Groups of AIMP3^fl/fl^ and UBC-Cre^ERT2^; AIMP3^fl/fl^ mice were given tamoxifen, and beginning from the date of injection, the mouse colony was monitored twice a week. Mice determined as moribund were euthanized and analysed under standard necropsy protocol. Survival curves were drawn using the Origin 8.0 program.

### Histology and immunohistochemistry

Mice were perfused with phosphate buffered saline, and organs were dissected and fixed in 10% neutral buffered saline overnight. Paraffin-embedded tissues were sectioned and stained with haematoxylin and eosin for routine histology. For immunohistochemical analysis, sections were deparaffinized and antigen retrieved in boiling sodium citrate buffer (pH 6.0) for 5 min. Sections were incubated in blocking buffer for 1 hr and with primary antibody in the same solution overnight. PBST (0.03% Triton X-100 in PBS) with 2% bovine serum albumin and 2% goat serum were used as a blocking buffer. Cleaved caspase-3 antibody (Cell signalling #9661, 1:200) and Dako system for rabbit was used for colorimetric development. Sections were dehydrated and mounted, and images were obtained using slide scanner (3D histech, panoramic midi) for histological analysis. For fluorometric detection, γH2AX (cell signalling, 1:200), RPA (Millipore NA19L, 1:200), Rad51 (abcam ab63801, 1:200), 53BP1 (Bethyl A300-272A, 1:1000), biotin conjugated Ki67 (affymetrix 1:200), Cy3-anti-rabbit antibody (Jackson immuno, 1:200), FITC anti-mouse antibody (Jackson immuno 1:200) and AF488 streptavidin (Jackson immuno, 1:200) was used. After counterstaining with Hoescht 33258 (1 μg/ml), tissues were mounted and observed under a fluorescence microscope. For BrdU labelling, BrdU was injected into pregnant females intraperitoneally 2 hr before euthanasia. E13.5 embryo heads were processed as described above. BrdU-containing sections were immersed in 4N HCl 20 min for antigen retrieval. Mouse anti-BrdU antibody (Sigma BU-33, 1:200) and FITC-anti-mouse IgG were used for staining.

### FACS analysis of haematopoietic cells

Mice were anesthetised and whole blood was obtained by retro-orbital bleeding. Two hundred microliters whole blood was used for the analysis of white blood cell quantification after RBC lysis. For pan-T, pan-B and pan-macrophage cell analysis, CD3ε-FITC (eBioscience, 1:800), CD45R-PE (eBioscience, 1:800) and CD11b-APC (Biolegend, 1:800) were incubated with cells for 30 min and were subsequently washed with FACS buffer. Suspended cells were analysed using FACS Calibur (Beckton Dickinson) and flowing software. Splenocytes were isolated by grinding spleen in a mesh screen (Sigma pore size 60 um) and suspending in RPMI media. Bone marrow was isolated by flushing the femur and tibia with RPMI media using a 26-gauge needle syringe. Cell suspensions were further analysed by FACS for the markers listed above.

### Generation of AIMP3^fl/fl^ MEFs and Ad5Cre virus treatment

Mouse embryonic fibroblasts were prepared from E13.5 AIMP3^fl/fl^ embryos. Primary cells were immortalized by introduction of SV40 T antigen. To delete AIMP3 in MEFs, cells were treated with Ad5Cre virus (titre 3 × 10^7^/ml) for one day and incubated a few days more to ensure deletion of AIMP3. As a control, Ad5GFP at the same titre was used. Ad5Cre and Ad5GFP virus were purchased from the viral vector core facility from the University of Iowa (USA). For γH2AX immunostaining, virus treated-cells were transferred and grown on coverslips. Cells were fixed with 4% paraformaldehyde for 10 min, permeabilised in 0.3% Triton X-100 for 10 min and stained with γH2AX antibody as described above. For counting γH2AX positive cells, approximately 200 cells from four independent fields were observed. For colony forming assay, virus treated-cells were plated at a density of 100/cm^2^ and grown for 8–10 days until colonies became visible. Colony numbers were counted after staining with crystal violet (2 μg/ml). All experiments were performed in triplicate.

### Immunoblotting

Tissues and cells were homogenized in RIPA buffer (150 mM NaCl, 50 mM Tris (pH 8.0), 0.1% SDS, 0.5% sodium deoxycholate). Total protein levels were measured using the BCA method (Thermo), and 40–80 μg of protein was loaded into 10–12% acrylamide gels. Proteins were transferred to PVDF membranes, blocked with 5% skim milk, and blotted with antibodies against AIMP3 (human atlas, 1:1000), γH2AX (cell signalling, 1:1000), phospho RPA(S4/S8) (Bethyl A300-245A, 1:1000), RPA (Millipore NA19L, 1:500), Chk1 (sc-8408, 1:500), phospho-Chk1 (cell signalling 2341, 1:1000), β-actin (Sigma AC-74, 1:10000), tubulin-α (sc-12462R, 1:1000), and hsp90 (Santa Cruz sc-7947, 1:1000) in 1% milk or 3% BSA in PBS. To detect multiple proteins, same samples were processed in parallel in different gels, or single transferred membranes were cut into pieces according to protein size for immunoblotting. Membranes were washed in PBST (0.03% Triton X) and blotted with HRP conjugated-antibodies against mouse or rabbit IgG (Thermo) in 5% skim milk. Membranes were developed using ECL reagent (Santa Cruz).

### Comet assay

Procedures are described in detail elsewhere^51^. Briefly, MEFs treated with GFP or Cre virus were suspended in 1% low melt agarose (37 °C) and 2 × 10^4^ cells were spread on a slide. Slides were alkaline-lyzed, electrophoresed and stained with propidium iodide (1 μg/ml) for imaging. More than 20 images per slide were analysed by OpenComet plug-in (v1.3) in ImageJ software.

### Homologous recombination activity assay

Procedures were described in detail elsewhere^52^. Briefly, U2OS cells were electroporated with pDRGFP or pimEJ5GFP vector (Addgene #26475 or #44026) and maintained in the medium (McCoy’s 5A) with puromycin (1 μg/ml) for clonal selection. A stable cell line was electroporated with siRNA (100 nM) including control, AIMP3, BRCA1, XRCC5, GARS (Dharmacon ON-TARGET plus siRNA SMARTpool) and two days later, 5 × 10^5^ cells were electroporated with pCBASceI plasmid (2 μg). The next day, cells were measured for the green fluorescence using FACS caliber (Beckton Dickinson). Total 10,000 cells were measured for each analysis. pDRGFP and pCBASceI were gifts from Maria Jasin (Addgene plasmids). For siRNA-mediated knock-down validation, cells were lyzed in Trizol (Gibco) and cDNA were synthesized from the 1 μg of total RNA using MMLV-RT (Clontech). Level of AIMP3, BRCA1, XRCC5 and GARS were measured by SyGr (Bio-rad) using AB7500 real time PCR. GARSf 5′-ccagaaactgcacaggggat-3′; GARSr 5′-gaggtagatgcggccaatga-3′; BRCA1f 5′ acagctgtgtggtgcttctgtg-3′; BRCA1r 5′-cattgtcctctgtccaggcatc-3′; AIMP3f 5′-ctggggagtactgcagaaga-3′; AIMP3r 5′-gacaaaaccagcgagacaca-3′; XRCC5f 5′-ccttcagccagactggagac-3′; XRCC5r 5′-cccaaatcctcgatttcaga-3′; GAPDHf 5′-tttggtcgtattgggcgcctg-3′; GAPDHr 5′-ccatgacgaacatgggggcat-3′.

### Methionine incorporation

Cells were trypsinized and resuspended in the methionine-free medium for 15 min. [^35^S]Methione was added to medium for 30 min for incorporation. Cells were precipitated with 10% TCA solution and radioactivity in the soluble and precipitate fraction were measured by liquid scintillation. Methionine incorporation was calculated as a percentage of precipitated radioactivity in total uptake.

### Ionizing radiation

After serial tamoxifen injection schedule, mice were anethesized and exposed to 7 Gy total body irradiation (TBI) in the Gammacell 3000 Elan. Mice were recovered and monitored daily for the gross phenotype. Mice to become moribund were sacrificed for histological analysis. For repair foci analysis, siRNA treated U2OS cells were exposed to 10 Gy irradiation and prepared for immunostaining or immunoblotting as described above.

No datasets were generated or analysed during the current study.

## Electronic supplementary material

Supplementary information
