# Supplementary material for: AIMP3 Deletion Induces Acute Radiation Syndrome-like Phenotype in Mice
Source: Sci Rep. 2018 Oct 9;8:15025. doi: 10.1038/s41598-018-33303-3 (PMC6177475; doi:10.1038/s41598-018-33303-3)
Supplement: Supplementary file 1 — Supplementary information [file 41598_2018_33303_MOESM1_ESM.pdf]

AIMP3 Deletion Induces Acute Radiation Syndrome-like Phenotype in Mice

Doyeun Kim[1], Sunmi Kim[2], Youngsun Oh[1], Songhwa Park[1], Yoon Jeon[2], Hongtae Kim[3], Ho Lee[2], and Sunghoon Kim[1]*

Fig S1

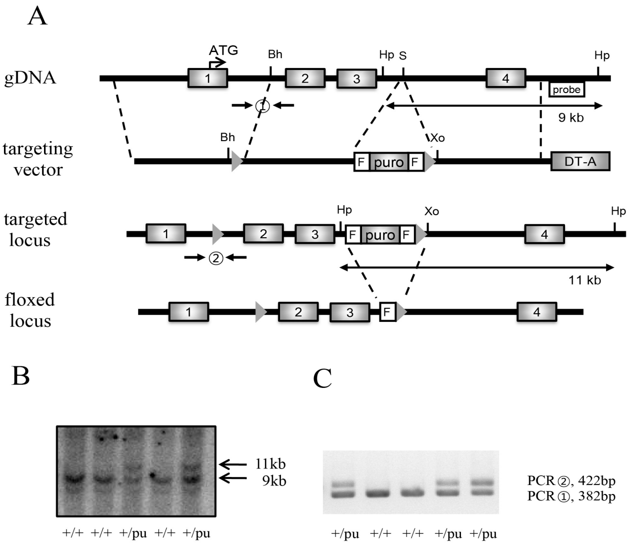
A
gDNA
ATG
Bh
Hp
S
Hp
1
2
3
4
probe
①
9 kb
targeting vector
Bh
Xo
F
puro
F
DT-A
targeted locus
Hp
Xo
Hp
1
2
3
F
puro
F
4
②
11 kb
floxed locus
1
2
3
F
4
B
11kb
9kb
+/+ +/+ +/pu +/+ +/pu
C
PCR②, 422bp
PCR①, 382bp
+/pu +/+ +/+ +/pu +/pu

Fig S2

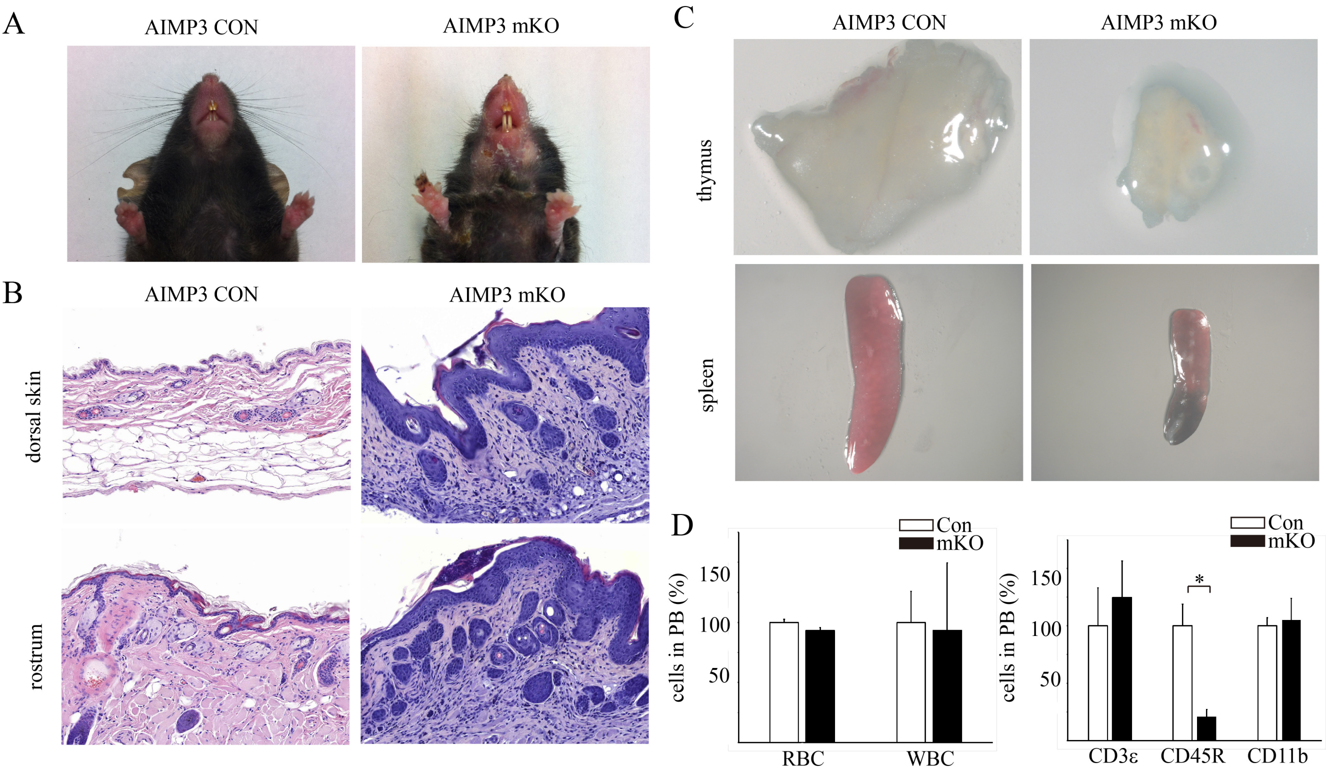
A
AIMP3 CON
AIMP3 mKO
B
AIMP3 CON
AIMP3 mKO
dorsal skin
rostrum
C
AIMP3 CON
AIMP3 mKO
thymus
spleen
D
Con
mKO
cells in PB (%)
150
100
50
RBC
WBC
Con
mKO
cells in PB (%)
150
100
50
*
CD3ε
CD45R
CD11b

Fig S3

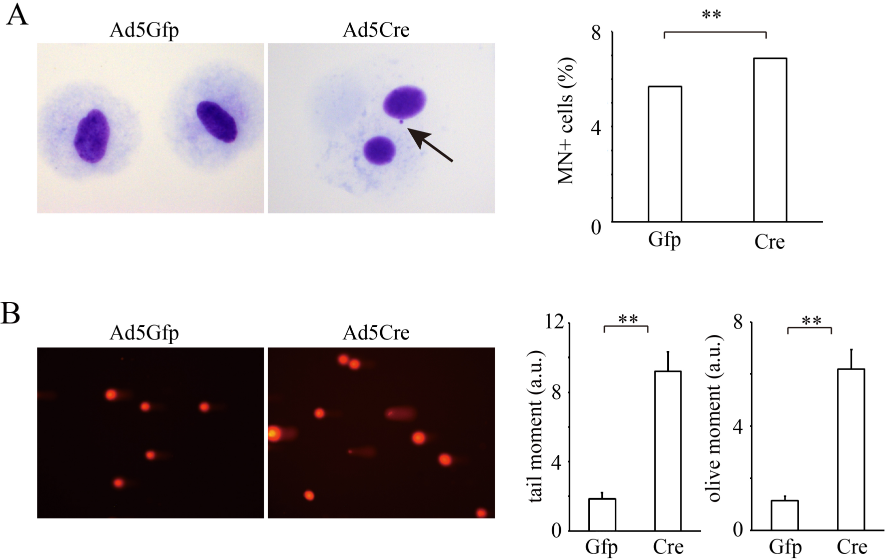

A
Ad5Gfp
Ad5Cre
MN+ cells (%)
8
4
0
**
Gfp
Cre
B
Ad5Gfp
Ad5Cre
tail moment (a.u.)
12
8
4
0
**
Gfp
Cre
olive moment (a.u.)
8
4
0
**
Gfp
Cre

Fig S4.

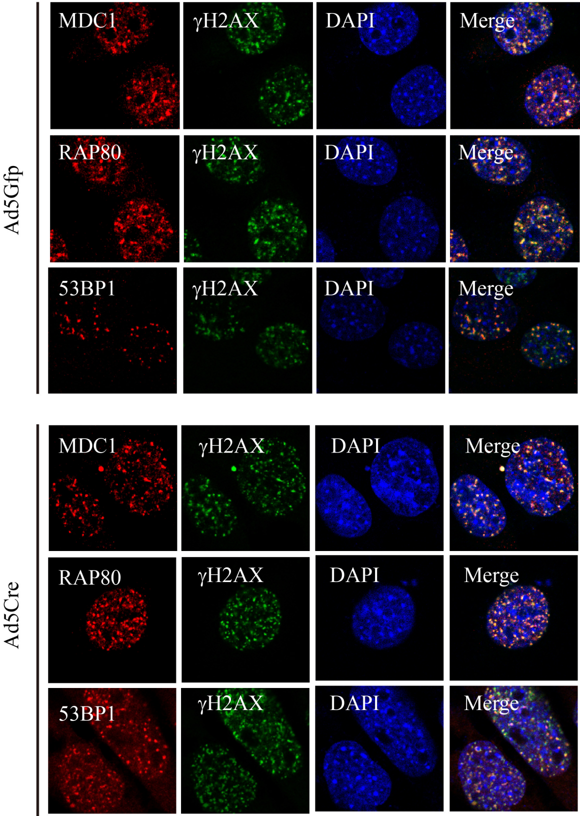
Ad5Gfp
MDC1
γH2AX
DAPI
Merge
RAP80
γH2AX
DAPI
Merge
53BP1
γH2AX
DAPI
Merge
Ad5Cre
MDC1
γH2AX
DAPI
Merge
RAP80
γH2AX
DAPI
Merge
53BP1
γH2AX
DAPI
Merge

Fig S5.

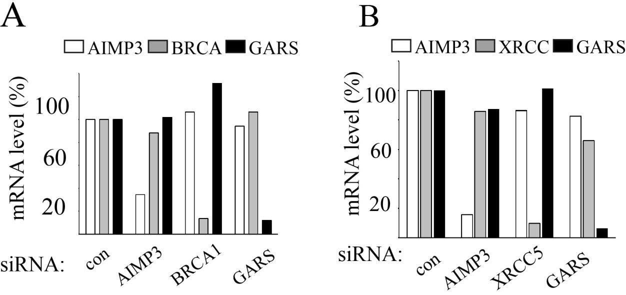
A
AIMP3
BRCA
GARS
mRNA level (%)
100
60
20
siRNA:
con
AIMP3
BRCA1
GARS
B
AIMP3
XRCC
GARS
mRNA level (%)
100
60
20
siRNA:
con
AIMP3
XRCC5
GARS

Fig S6.

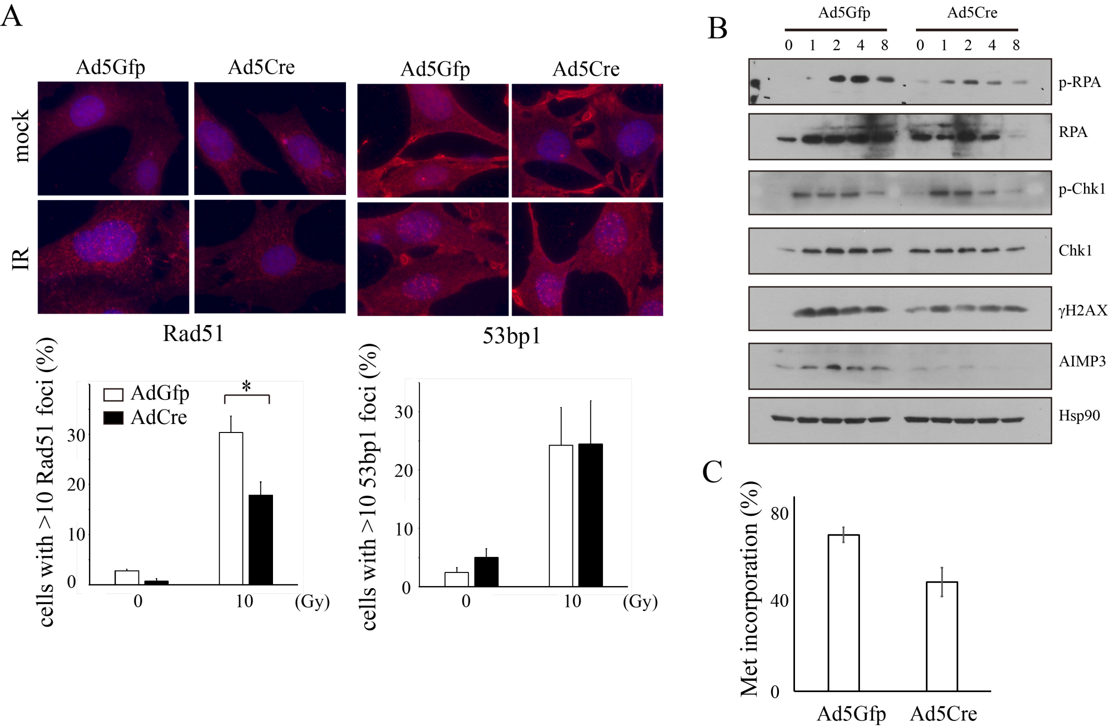
A
Ad5Gfp
Ad5Cre
Ad5Gfp
Ad5Cre
mock
IR
Rad51
53bp1
cells with >10 Rad51 foci (%)
AdGfp
AdCre
*
0
10
20
30
0
10
(Gy)
cells with >10 53bp1 foci (%)
0
10
20
30
0
10
(Gy)
B
Ad5Gfp
Ad5Cre
0 1 2 4 8
0 1 2 4 8
p-RPA
RPA
p-Chk1
Chk1
γH2AX
AIMP3
Hsp90
C
Met incorporation (%)
0
40
80
Ad5Gfp
Ad5Cre

Supplemental Figure legend

Figure S1. Targeting of the AIMP3 locus

(A) Schematic representation of the AIMP3 locus, the targeting vector and the targeted loci. Exon 1-4, southern blot probe, translation start site (ATG), puromycin cassette (puro), diphtheria toxin A chain gene (DT-A), FRT sequence (F), loxP sequence (triangle) and restriction sites (Bh, BamHI; Hp, HpaI; S, SalI) are shown. The circle number with arrows notes the primer binding site for genotyping analysis.

(B) A southern blot analysis of a representative AIMP3$^{+/puro}$ ES cell clones is shown. For HpaI digestion, the bands representing wild-type and mutant alleles are ~9.0 kb and ~ 11.0 kb, respectively.

(C) PCR analyses with genomic DNA extracted from ES cell clones. Upper band (2) and lower band (1) derives from targeted and wild type locus, respectively.

Figure S2. Gross phenotype of AIMP3 mKO mice

(A) Representative images of rostrum and forelimb of AIMP3 mKO mouse. Hair loss and skin thickening is frequently observed.

(B) Histology of dorsal (top) and rostral (bottom) skin from AIMP3 CON or AIMP3 mKO mice. AIMP3 mKO mice show thickening of epidermis and enriched macrophage in the dermis.

(C) Representative gross image of thymus and spleen from AIMP3 CON or AIMP3 mKO.

(D) Peripheral blood analysis. Red blood cells (RBC) or white blood cells (WBC) were counted from the whole blood (left). After RBC lysis, WBC were analyzed by CD3ε, CD45R, or CD11b marker expression.

Figure S3. Spontaneous double strand breakage in AIMP3 KO MEF cells

(A) Chromosome spread was prepared from MEF cells treated with Ad5Gfp or Ad5Cre. Nucleus with micronuclei (arrows) were counted from more than 1000 nuclei. Statistical significance was calculated by Fisher's exact method (**$p<0.01$).

(B) The occurrence of DNA breakage was analyzed by neutral comet assay in virus-treated AIMP3 fl/fl MEF cells. Representative images are shown from 20-25 images from randomly selected fields. Olive

and tail moment was calculated using the Open comet plugin in the ImageJ program. Statistical significance was calculated by Student's t-test (**$p<0.01$).

Figure S4. H2AX recognition foci formation in AIMP3 KO MEF cells

MEF cells were irradiated with 10 Gy and immunostained with indicated antibodies at 3 hours after irradiation. MDC1, RAP80 and 53BP1 foci formation was not decreased in AIMP3 KO MEF cells.

Figure S5. Effect of siRNA in U2OS cells

Total RNA from siRNA treated-cells were isolated and the level of AIMP3, BRCA1 and GARS mRNA (A) or AIMP3, XRCC5 and GARS mRNA (B) was measured by real-time PCR analysis. Relative amount to the siCON treated-cells were indicated respectively.

Figure S6. Reduced repair foci formation in AIMP3 KO MEF cells

(A) Measurement of foci formation by immunostaining using Rad51 (left) or 53BP1 (right) antibodies. SiRNA-treated U2OS cells were irradiated (10Gy) and foci was observed at 2 and 4 (hr) after irradiation respectively. immunostained with indicated antibodies. Nucleus with more than 10 foci was counted as a positive and quantitated as percentage among >200 cells (* $p<0.001$ by two-way ANOVA with Bonferroni's posttests).

(B) Measurement of RPA protein level in AIMP3 KO MEF cells. Ad5Gfp or Ad5Cre treated AIMP3 fl/fl cells were irradiated and total protein were isolated at indicated time point. Same lysates were processed in parallel in different gels for immunoblotting. Single transferred membrane was cut into pieces and used for the detection of protein pairs; p-RPA and hsp90, p-Chk1 and AIMP3, Chk1 and RPA. Lysates were immunoblotted with indicated antibodies. Hsp90 was used as a loading control.

(C) Protein synthesis rate in the Ad5Gfp or Ad5Cre-treated MEF cells was measured by [^{35}S] methionine incorporation to the total protein. The results were obtained from triplicated samples.

Error bar indicates standard error.
